# Folic Acid-Decorated pH-Responsive Nanoniosomes With Enhanced Endocytosis for Breast Cancer Therapy: *In Vitro* Studies

**DOI:** 10.3389/fphar.2022.851242

**Published:** 2022-04-20

**Authors:** Tahereh Rezaei, Mehdi Rezaei, Sara Karimifard, Farzaneh Mahmoudi Beram, Mohammad Sedigh Dakkali, Maryam Heydari, Soheil Afshari-Behbahanizadeh, Ebrahim Mostafavi, Dmitry Olegovich Bokov, Mohammad Javed Ansari, Bahareh Farasati Far, Iman Akbarzadeh, Chaiyavat Chaiyasut

**Affiliations:** ^1^ General Physician, Shiraz University of Medical Sciences, Shiraz, Iran; ^2^ Department of Cardiology, Fars-Iranian Heart Association, Fars Society of Internal Medicine, Shiraz, Iran; ^3^ Stem cells Research Center, Tissue Engineering and Regenerative Medicine Institute, Islamic Azad University, Central Tehran Branch, Tehran, Iran; ^4^ Department of Chemical and Petrochemical Engineering, Sharif University of Technology, Tehran, Iran; ^5^ School of Medicine, Iran University of Medical Sciences, Tehran, Iran; ^6^ Department of Cell and Molecular Biology, Faculty of Biological Science, University of Kharazmi, Tehran, Iran; ^7^ Faculty of Veterinary Medicine, Islamic Azad University, Garmsar Branch, Garmsar, Iran; ^8^ Stanford Cardiovascular Institute, Stanford University School of Medicine, Stanford, CA, United States; ^9^ Department of Medicine, Stanford University School of Medicine, Stanford, CA, United States; ^10^ Institute of Pharmacy, Sechenov First Moscow State Medical University, Moscow, Russia; ^11^ Laboratory of Food Chemistry, Federal Research Center of Nutrition, Biotechnology and Food Safety, Moscow, Russia; ^12^ Department of Pharmaceutics, College of Pharmacy, Prince Sattam Bin Abdulaziz University, Al-kharj, Saudi Arabia; ^13^ Department of Chemistry, Iran University of Science and Technology, Tehran, Iran; ^14^ Innovation Center for Holistic Health, Nutraceuticals, and Cosmeceuticals, Faculty of Pharmacy, Chiang Mai University, Chiang Mai, Thailand

**Keywords:** 5-FU, breast cancer, endocytosis, folic acid, hyaluronic acid, niosome

## Abstract

Breast cancer is the most common invasive cancer in women and the second leading cause of cancer death in women after lung cancer. The purpose of this study is a targeted delivery toward *in vitro* (on MCF7 and 4T1 breast cancer cell lines) through niosomes-based nanocarriers. To this end, different bioactive molecules, including hyaluronic acid (HA), folic acid (FA), and polyethylene glycol (PEG), were used and compared for surface modification of niosomes to enhance endocytosis. FA-functionalized niosomes (Nio/5-FU/FA) were able to increase cell cytotoxicity and reduce cell migration and invasion compared to PEG-functionalized niosomes (Nio/5-FU/PEG), and HA-functionalized niosomes (Nio/5-FU/HA) groups in MCF-7 and 4T1 cell lines. Although the Nio/5-FU/PEG and Nio/5-FU/HA demonstrated MCF7 cell uptake, the Nio/5-FU/FA exhibited the most preponderant endocytosis in pH 5.4. Remarkably, in this study 5-FU loaded niosomes (nonionic surfactant-based vesicles) were decorated with various bioactive molecules (FA, PEG, or HA) to compare their ability for breast cancer therapy. The fabricated nanoformulations were readily taken up by breast cancer cells (*in vitro*) and demonstrated sustained drug release characteristics, inducing cell apoptosis. Overall, the comprehensive comparison between different bioactive molecules-decorated nanoniosomes exhibited promising results in finding the best nano formulated candidates for targeted delivery of drugs for breast cancer therapy.

## Graphical Abstract

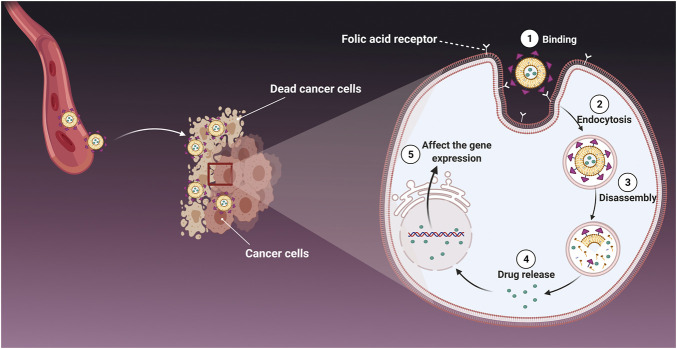

## 1 Introduction

Breast cancer is a disease in which cells in the breast grow out of control. To date, chemotherapy, radiation therapy, and surgery are used to treat breast cancer (Sarhadi et al., 2020; Kumar et al., 2021; Garg et al., 2022; Sahrayi et al., 2022). The poor efficacy of chemotherapy and its adverse effects on healthy cells (i.e., bone marrow suppression, hair loss, gastrointestinal reactions) are the main drawbacks of these conventional therapies (Molani et al., 2019; Molani et al., 2020). To bypass these drawbacks, targeted nano-delivery approaches, such as niosomes, have been extensively studied in an attempt to create a breakthrough in the bottleneck of chemotherapeutic treatment of breast cancer (Tavakoli et al., 2021).

Recently, colloidal drug delivery such as niosomes has been used to transport and deliver bioactive molecules to the tumor site (Dwivedi et al., 2020). Niosomes are microscopical lamellar structures composed of cholesterol. They are nonionic surfactants of the alkyl/dialkyl polyglycerol ether class, with subsequent hydration in an aqueous medium. Although niosomes are structurally similar to liposomes in having a bilayer, the materials employed to prepare niosomes to improve their stability enable them to load hydrophilic and lipophilic drugs. Furthermore, niosomes are one of the lipid-based nanocarriers that are more cost-effective and have more accessible storage of the nonionic surfactants than phospholipid-containing liposomes (Rajera et al., 2011; Marianecci et al., 2014; Moghassemi and Hadjizadeh, 2014). In addition, niosomes have more features and benefits than liposomes (e.g., chemical stability, better biocompatibility, longer storage life, and better handling) (Bartelds et al., 2018). Niosomes have been used to deliver hormones, antigens, antimicrobial peptides, and chemotherapeutic drugs. Some niosomes are pH-responsive materials based on their composition. An acidic pH enhances the hydrolysis of surfactant molecules in the periphery of a niosome, resulting in a burst release of loaded biomolecules (Moghtaderi et al., 2021). The surface of niosomes is frequently functionalized with biomolecules to enhance their endocytosis into cancer cells, especially those that contain over-expressed receptors on their cell membranes (Mohammadinejad et al., 2020). For instance, polyethylene glycol (PEG), a highly water-soluble, biocompatible, non-toxic, non-immunogenic, and non-antigenic polymer, has been used extensively as a surface modifier synthesis of niosomes (Alemi et al., 2018). Hyaluronic acid (HA) is another biological polymer that has been employed because the receptor for HA, CD44, is overexpressed in the tumor microenvironment (Mohammadinejad et al., 2020; Delfi et al., 2021; Pan et al., 2021). It is over-expressed on the surface of different carcinoma cells, including breast cancer cells (Ji et al., 2020). Another bioactive molecule commonly used to functionalize niosomes is folic acid (FA). Folic acid is an anionic molecule that does not diffuse across biological membranes quickly. This biomolecule meets the demand of cancer cells for folate by targeting overexpressed FA receptors on their cell surface (Kumar et al., 2019).

**FIGURE 1:**
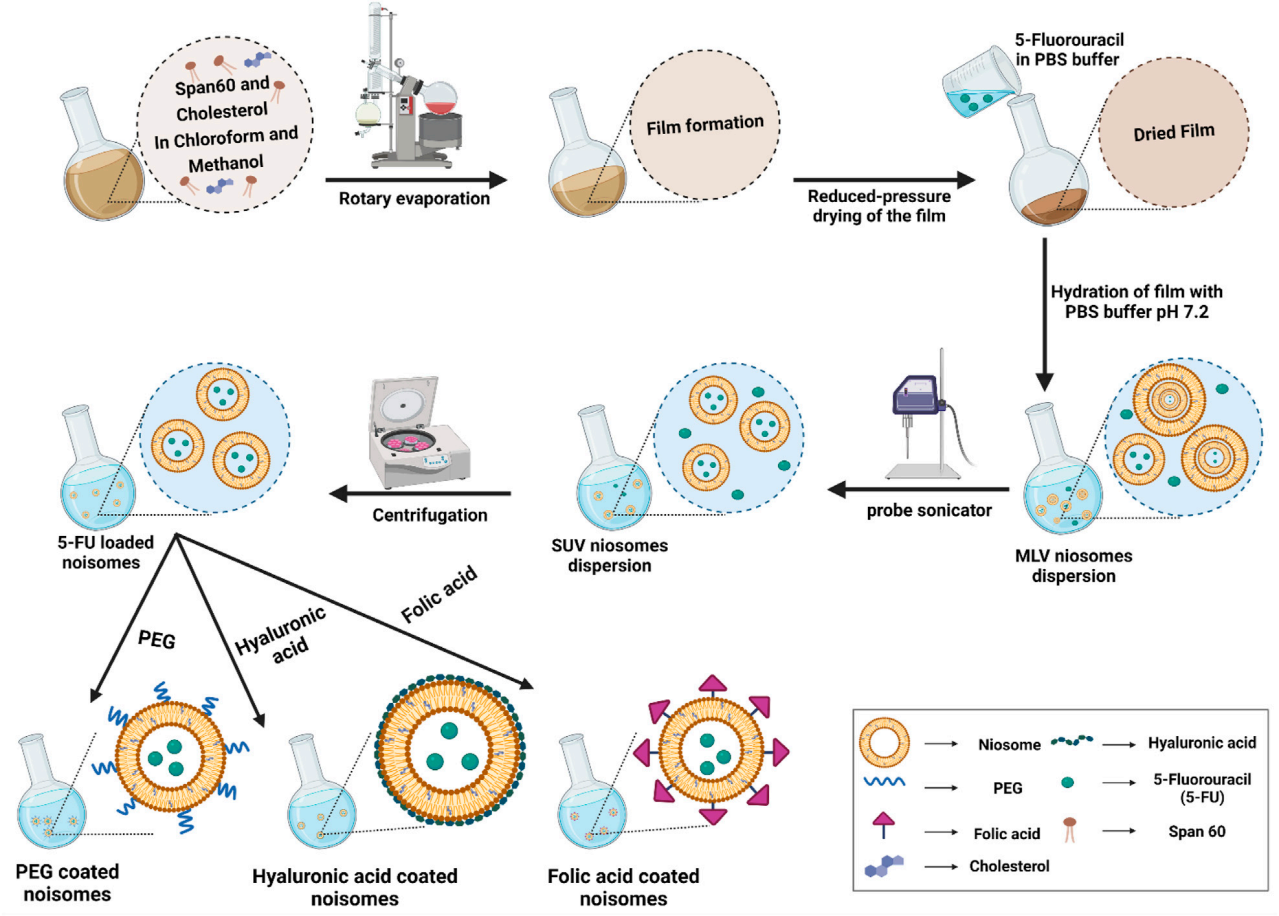
The preparation and characterization of functionalized niosomes by the thin layer hydration method. MLV: multilamellar vesicles; SUV: small uni-lamellar vesicles.

Despite the advantages of existing anticancer drugs such as 5-Fluorouracil (5-FU), their use is limited due to poor penetration into tumor tissues, high metabolic rate, unwanted side effects, and gradual development of tumor cell resistance. Thus, the main goal of this study is to improve the targeted uptake and reduce the off-target toxicity of drugs employed in breast cancer treatment. To achieve this, the niosomes were first loaded with 5-FU as a model drug. Then they were decorated with HA, PEG or FA by the thin-layer hydration method. The modification was designed to increase the endocytosis of the niosomes in cancer cells (Figure 1), and we compared the stability, drug release, and cell cytotoxicity of different modified niosomes with each other. The optimized 5-FU-loaded niosomes were characterized in morphology, size, polydispersity index and encapsulation efficacy. Since 5-FU-loaded niosomes are easily taken up by breast cancer cells and demonstrate sustained drug release characteristics, our study’s main objective was to fabricate and compare 5-FU-loaded niosomes (nonionic surfactant-based vesicles) that were decorated with FA, PEG, or HA for breast cancer therapy. The stability of the nanoformulations was determined by physical change and the percentage of drugs remaining in different conditions for up to 30 days. Cell cytotoxicity, apoptosis, and flow cytometry of the nanocarriers were evaluated.

## 2 Materials and Methods

### 2.1 Materials

Fluorouracil (5-FU), Span^®^ 60 (sorbitan monostearate) and cholesterol were purchased from Millipore Sigma (Burlington, MA United States) and used without further purification. Chloroform, methanol, polyethylene glycol 2000 (PEG 200), hyaluronic acid (HA), FA-derivatized mPEG2000-DSPE (FA-PEG2000-DSPE), dimethyl sulfoxide (DMSO), dialysis membrane (MWCO 12,000 Da), sodium dodecyl sulfate (SDS), phosphate-buffered saline (PBS) and Amicon (Ultra-15-Membrane, MWCO 30,000 Da) were acquired from Merck Chemical Co. (Darmstadt, Germany). The MCF10A, MCF7 and 4T1 cell lines were purchased from Pasteur Institute Cell Bank (Tehran, Iran). Medium RPMI-1640 (Dulbecco’s Adjusted Eagle Medium), formaldehyde, trypsin-EDTA, Trypan blue, fetal bovine serum (FBS), phosphate-buffer, 3-(4,5-dimethylthiazol-2-yl)-2,5-diphenyltetrazolium bromide (MTT) and penicillin/streptomycin (PS) 100 X were acquired from Gibco, ThermoFisher Scientific (Waltham, MA, United States). 1X binding buffer and annexin V-FITC flow cytometry kit was obtained from Affymetrix Biosciences, ThermoFisher Scientific. Dialysis membrane (MWCO 12,000 Da), Nile red, and Coumarin 6 were purchased from Millipore Sigma. DCFDA/H2DCFDA—Cellular Reactive Oxygen Species (ROS) Assay Kit was obtained from Abcam Company (Cambridge, United Kingdom).

### 2.2 Optimization of Niosomal Formulations Using Response Surface Methodology

The response surface methodology (RSM) was used for the optimization of niosomal formulations via a central composite design (CCD) approach. To investigate the relationship between a set of independent variables and the dependent variables by fitting the data using a polynomial equation, two numerical parameters (Span^®^ 60 and cholesterol) were selected to study the impact of their concentration on niosomal particle size (nm), polydispersity index (PDI), entrapment efficacy percentage (EE %) and percentage drug release in 24 h. The polynomial equation was obtained using Design-Expert software (Version 10, Stat-Ease, Inc., Minneapolis, MN, United States). Comparisons were made between the experimental data and the predicted responses. Using the point prediction method, the optimal formulation was chosen for further study (Khanal et al., 2021). Span^®^ 60 and cholesterol were chosen based on their low, medium and high levels (Supplementary Table S1).

### 2.3 Synthesis of Niosomal Formulations

Niosomes loaded with 5-fluorouracil (Nio/5-FU) were prepared using the thin-film hydration method (Akbarzadeh et al., 2020b; Moghtaderi et al., 2021). Briefly, cholesterol and Span^®^ 60 were dissolved in 9 ml of chloroform/methanol (2:1; v/v). The solution was transferred to a 50 ml round bottom flask. The organic solvent was evaporated under vacuum using a rotary evaporator (Heidolph Instruments, Schwalbach, Germany) at 60°C and 150 rpm for 30 min, until a thin dried film was formed in the bottom of the flask. The 5-FU-loaded niosome was formed by hydration of the thin film at 60°C using PBS containing 10 mg of 5-FU (1X, 10 ml, pH 7.4). Subsequently, the reactants were dried at 60°C and 150 rpm for 30 min to obtain dried specimens. Homogenized samples were prepared using an ultrasonic processor (UP50H compact laboratory homogenizer, Hielscher Ultrasonics, Teltow, Germany) to ensure that the Nio/5-FU had an optimal size distribution (Figure 1). The non-entrapped 5-FU was separated from the entrapped 5-FU with the ultrafiltration method. The samples were kept at 4 C to characterize the different niosomal formulations listed in Supplementary Table S2.

### 2.4 Surface Functionalization of the Optimized Niosomal Formulation

The above prepared Nio-5-FU suspension was added into PEG solution at 0.5 mg/ml concentration, stirred for 15 min at room temperature, and left overnight. The sonicated solution was homogenized for 10 min at 12,000 rpm (Alemi et al., 2018). Niosomes coated with HA (Nio/5-FU/HA) were synthesized by titrating 20 ml of PBS containing 0.1% (w/v) HA into 0.2 g of 5-FU loaded niosomes (Nio/5-FU). The optimum Nio-5-FU was then centrifuged (40,000 rpm, 60 min), and the pellet continued under stirring were added dropwise into the HA suspension. The resulting preparations were stirred up to 24 h at room temperature. The hyaluronic acid solution was prepared by stirring the weighed HA powder in deionized water and adding it to the hydration process. (Zeng et al., 2016). Niosomes coated with folic acid (Nio/5-FU/FA) were synthesized by dissolving Span^®^ 60, cholesterol and FA-PEG2000-DSPE in 9 ml of chloroform/methanol (2:1; v/v), subsequently, the solvent was removed by rotary evaporation at 160 rpm, at 60°C for 30 min, forming a dried thin film residue. Nio/FA loaded with 5-FU was formed by hydration of the thin film at 60°C using PBS containing 10 mg of 5-FU (1X, 10 ml, pH 7.4). Subsequently, the reactants were dried at 60°C and 150 rpm for 30 min to obtain dried specimens. The samples were sonicated for 5 min to obtain a uniform size distribution, and finally, they were stored at 4°C until further use (Figure 1).

### 2.5 Physical Characterization of Niosomal Formulations

The particle size, Zeta Potential, and polydispersity index (PDI) of the niosomal formulations were determined at 25°C using a benchtop dynamic light scattering/electrotrophoretic light scattering system (Zetasizer Nano S90, Malvern Panalytical Ltd., Malvern, United Kingdom). The morphology of the specimens was observed using transmission electron microscopy (TEM). A drop of niosomal formulation was placed on a carbon-coated copper grid and stained with a 1% phosphotungstic acid. The stained niosomes were imaged with TEM (Model EM900, Zeiss Microscopy, Jena Germany) at 100 kV (Muzzalupo et al., 2005; Sohrabi et al., 2016).

### 2.6 Entrapment Efficacy

The niosomal formulations were ultra-filtered (Eppendorf^®^ 580R centrifuge, Hamburg, Germany) with an Amicon Ultra-15-membrane at 4,000 rpm for 20 min at 4°C. The non-entrapped 5-FU was separated from the entrapped 5-FU to determine the percentage of entrapment efficacy (EE%). The free drug concentration was calculated at 266 nm using UV-Visible light spectrophotometer (UV-1700 PharmaSpec, Shimadzu, Kyoto, Japan). The following equation was used to calculate EE% (Rochani et al., 2016).

 EE%=[(A−B)/A]×100%

where (A) is the initial 5-FU concentration for the niosomal preparation and (B) is the concentration of non-entrapped 5-FU after centrifugation.

### 2.7 *In vitro* Drug Release

For evaluation of drug release, 2 ml of each sample was placed in a semipermeable acetate cellulose dialysis bag (MWCO 12 kDa). The latter was immersed in 50 ml of PBS-SDS (0.5% w/v; release medium). The assembly was agitated at 50 rpm using a magnetic stirrer in various pH conditions (7.4 and 5.4) at 37°C for 72 h. At a specific time (1, 2, 4, 8, 24, 48, and 72 h), 1 ml of the release medium was withdrawn and replenished with the same volume of fresh PBS-SDS (Akbari et al., 2020). The amount of drug released at predetermined intervals was estimated at 238 nm using an ultraviolet light spectrophotometer. The test was repeated using a free drug as control, in which the drug concentration was equivalent inside and outside the dialysis bag.

Different mathematical models were used to evaluate the release kinetics of 5-FU from the samples: the Korsmeyer-Peppa model (log cumulative % drug release vs. log time), the Higuchi model (cumulative % drug release vs. square root of time), first-order model (cumulative % drug remaining vs. time) and zero-order model (cumulative % drug release vs. time) (Dash et al., 2010a; Kamboj et al., 2014; Shaker et al., 2015; Alemi et al., 2018). The correlation coefficient values (r) obtained by regression of the plots derived from the above models were used to calculate the linear curve. The zero-order model is dependent on drug dissolution, which describes the system where the drug release rate is independent of the concentration. The first-order rate equation describes drug release where the rate of drug release depends on its concentration (Dash et al., 2010a). There is a direct relationship between the amount of drug released from a matrix system and the square root of time in the Higuchi and the Korsmeyer-Peppa models (Higuchi, 1963; Korsmeyer et al., 1983). An initial release of 60% was adequate to determine the most suited model for drug release (Bettini et al., 2001).

### 2.8 FTIR Spectroscopy

A perusal of molecular interaction between ciprofloxacin and niosomes, Fourier Transform Infrared Spectroscopy (FTIR) (Spectrum Two, U.S.A.) was used. For this test, lyophilized samples were mixed separately in KBr and the pellets formed by placing the samples in a hydraulic press. FTIR analyses were accomplished in the scanning range of 4000 to 400 cm^−1^ in a constant resolution of 4 cm^−1^ and at room temperature.

### 2.9 Stability

The Nio/5-FU, Nio/5-FU/PEG, Nio/5-FU/FA, and Nio/5-FU/HA samples were stored at 25 ± 1°C or 4 ± 1°C, at 60 ± 5 % relative humidity for 1 month. After storage, the samples were analyzed for particle size, PDI, and percentage of drug remaining for evaluation of the stability of the formulations.

### 2.10 Culture of MCF7 and 4T1 Cell Lines

The human mammary carcinoma cell line MCF7 and the mouse mammary carcinoma cell line 4T1 were cultured at 37°C in an atmospheric supplemented with 5% CO_2_. The culture medium consisted of RPMI-1640 fresh medium supplemented with 10% FBS and 1% penicillin/streptomycin (complete growth medium). The medium was aspirated after the cells reached 85-95% confluence. The cell monolayer was detached using 0.25% (w/v) trypsin-EDTA. The detached cells were resuspended in a complete growth medium, labeled trypan blue and counted with a hemocytometer.

### 2.11 Cell Viability

Different concentrations of 5-FU, Nio/5-FU, Nio/5-FU/PEG, Nio/5-FU/HA, or Nio/5-FU/FA (0.25, 0.5, 1, 2, 4 and 8 μg/ml) were added to the cultured MCF7 and 4T1 cells and incubated for 48 h. For control, different dilutions of empty niosomes (Nio) were added to a non-tumorigenic epithelial cell line (MCF10A) and incubated for 48 h. To evaluate cell proliferation, the three types of cells were individually incubated with 0.5 mg/ml of MTT for 4 h to reduce the colorless tetrazolium dye MTT to insoluble formazan, which has a purple color. Formazan was dissolved in 100 μL of DMSO for colorimetric determination of the oxidoreductase enzymatic activity. Quantification was performed using the formula:

Percentage cell viability (%) = Optical Density 570-630 treatment/Optical Density 570-630 control × 100%

### 2.12 Flow Cytometry

The flow cytometry assay was used to evaluate the percentage of apoptotic MCF7 and 4T1 cells. Cultured cells were treated with Nio, 5-FU, Nio/5-FU, Nio/5-FU/PEG, Nio/5-FU/HA or Nio/5-FU/FA for 48 h. The Apoptosis and Necrosis Quantitation Kit was used to stain apoptotic cells with green fluorescence and necrotic cells with red fluorescence for examination by flow cytometry. The cells were rinsed twice with PBS and suspended in 1X binding buffer (5 × 10^5^ cells/ml). Cells were stained sequentially in annexin V-FITC (green fluorescence) and propidium iodide (red fluorescence) and analyzed with a benchtop flow cytometer (FACSCalibur, D Biosciences, Franklin Lakes, NJ, United States).

### 2.13 Reactive Oxygen Species

The culture of both types of cancer cells was performed using coverslips placed inside a 4-well plate containing RPMI-1640 medium. After culturing, the cells were incubated with 2′,7′-dichlorodihydrofluorescein diacetate (H_2_DCFDA) at 37°C for 30 min. The cells were washed with buffered saline and subsequently treated with Nio, 5-FU, Nio/5-FU, Nio/5-FU/PEG, Nio/5-FU/HA or Nio/5-FU/FA for 24 h. The cells were washed in PBS before being incubated for 30 min at 37°C with 80 mM H2DCFDA. A microplate reader was used to quantify the fluorescence intensity at 530 nm.

### 2.14 Confocal Laser Scanning Microscopy

1 × 10^5^ cells were seeded for 24 h in plates containing RPMI-1640 medium supplemented with 10% FBS. Nile red was used as a model molecule (500 μL) and loaded into the niosomes. The unloaded stain was removed by dialysis (MWCO 12 kDa). The Nile red-loaded niosomes (50 μg/ml) were incubated with cells for 3 h. The cancer cells were then washed with PBS, fixed with 4% formaldehyde, stained with coumarin-6 (green fluorescence) for 15 min, and examined with a confocal laser scanning microscope (TCS SP5, Leica Microsystems, Wetzlar, Germany).

### 2.15 Statistical Analysis

Statistical analysis and curve fitting were performed using GraphPad Prism software version 8 (GraphPad Software, Inc., San Diego, CA, United States). Data from three independent experiments were expressed as means ± standard deviations. Statistical significance was determined with a one-way analysis of variance after validating the normality and homoscedasticity of the data sets. For all analyses, statistical significance was pre-set at *α* = 0.05. Central composite design (CCD) was performed using Design-Expert software version 10 (Stat-Ease Inc., Minneapolis, MN, United States).

## 3 Results and Discussion

### 3.1 Characterization

#### 3.1.1 FTIR Spectroscopy

The empty noisome exhibits stretching peaks for C-O, C=O, and C-H at 1,125, 1747, and 2,900 cm^−1^, respectively. Additionally, it exhibits a carbonyl bond at 1,625 cm^−1^ and a -NH stretching vibration at 3,100–3,400 cm^−1^, indicating that noisome was successfully formed. Following drug loading, the carbonyl group shifted to 1,614 cm^−1^ and the stretching amide group to 3,095 cm^−1^, indicating the presence of 5-fu in the niosome structure. Following PEGylation of the structure of the drug-loaded niosome, the C-H group corresponds to the PEG structure visible in the final formulation’s 1,300 cm^−1^ region, confirming the structure’s PEGylation. After adding hyaluronic acid to the drug-loaded niosome, a peak at 1,655 cm^−1^ corresponding to the amide group appears in the region, confirming the successful incorporation of HA into the final structure. The final stage of the work involved adding folic acid to the final structure (drug-loaded niosome), which resulted in the appearance of the C-N stretching group in the 1,015 cm^−1^ and 1,279 cm^−1^ regions, confirming the presence of folic acid in the final formulation.

#### 3.1.2 Particle Size

**FIGURE 2:**
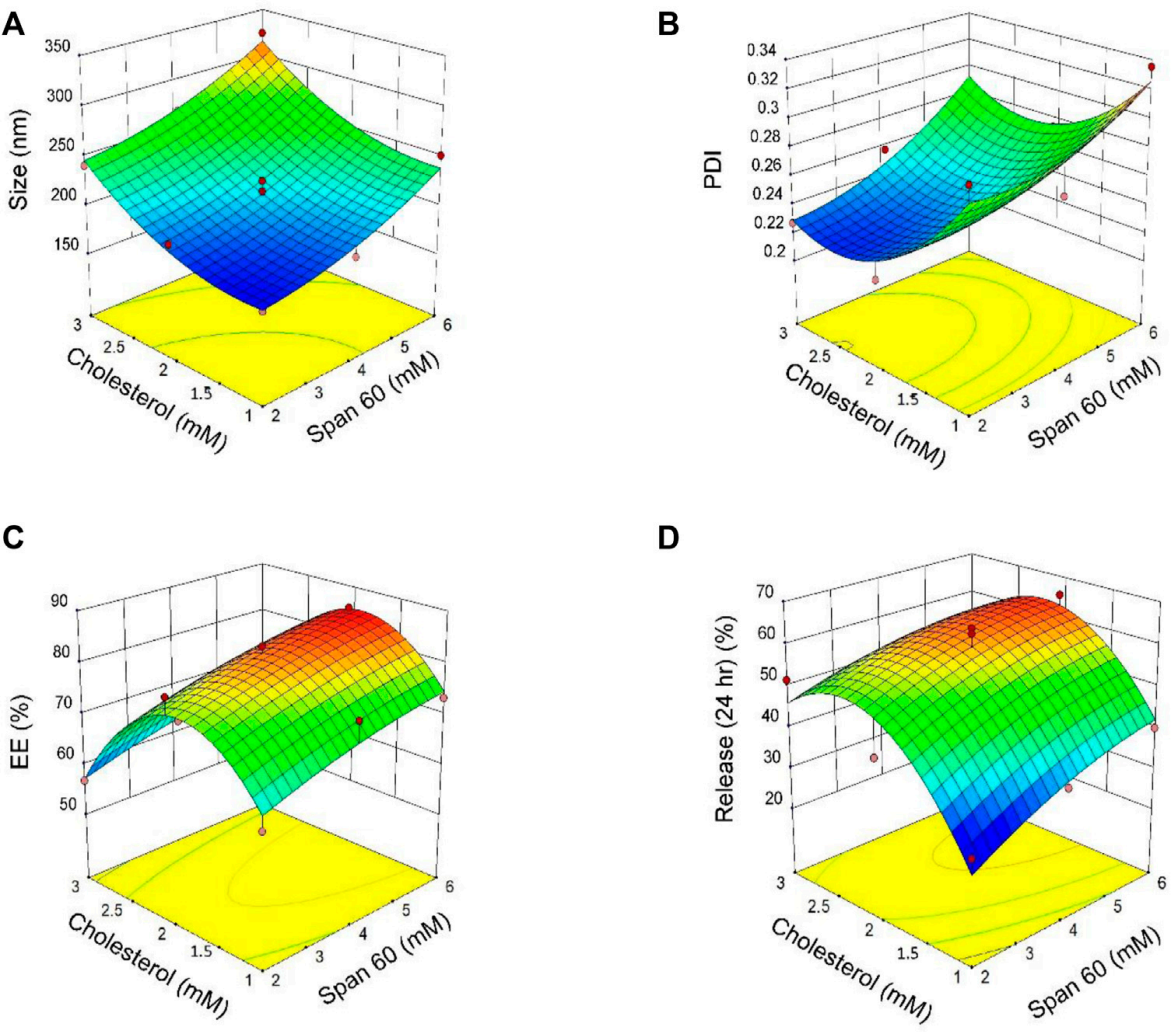
3D plots of the results derived from the central composite design for size **(A)**, polydispersity index [PDI; **(B)**], encapsulation efficacy [EE; **(C)**] and release **(D)** as a function of the parameters (concentrations of Span^®^ 60 and cholesterol).

Eleven formulations were prepared to evaluate the interactions between two independent variables: the surfactant sorbitan monostearate (Span® 60; Millipore Sigma, Burlington, MA, United States) and the cholesterol concentration at three levels. Different molar ratios of surfactant: cholesterol were used for response optimization. Responses to the experimental design were analyzed using multiple linear regression analyses to develop model equations. The responses derived experimentally from the independent variables were subsequently compared with the predicted values generated by the model equations. A significant coefficient was applied to evaluate models. Response values derived from the experiments mentioned above are summarized in (Supplementary Table S2). Based on Supplementary Table S2, particle size decreased from 324.7 nm (surfactant: cholesterol molar = 6: 3) to 174.2 nm (surfactant: cholesterol molar = 2:1). The results of Supplementary Table S3 and Supplementary Table S4 showed that “span60 and cholesterol have a significant effect on particle size (*p < 0.05*). The curvature of the 3-D and contour plots was employed to indicate the interaction and effects of two independent variables (Span^®^ 60 and cholesterol). Figures 2A–D describes the result of 3D plots for size (A), polydispersity index (PDI; B), encapsulation efficacy (EE; C), and release (D). According to Figure 2A, Span^®^ 60 and cholesterol have the same effect on particle size.

#### 3.1.3 Polydispersity Index

The PDI of all niosomes formulations obtained by central composite design (CCD) ranged from 0.214 to 0.335 (Supplementary Table S2). Based on Supplementary Table S3 both independent variables (Span^®^ 60 and cholesterol) have a significant effect on PDI (*p <* 0.05). According to Supplementary Table S4 for the regression, and Figure 2B for 3D contour plots, the independent variable Span^®^ 60 has a positive effect on PDI and PDI decreased with increasing cholesterol concentrations.

#### 3.1.4 Entrapment Efficacy

The *p* values of the encapsulation efficiency are summarized in Supplementary Table S3 and the regression equation for EE% is presented in Supplementary Table S4. The results show Span^®^ 60, significantly affected the percentage of entrapment efficacy (EE%; *p <* 0.05) while cholesterol had no significant effect on EE% (*p >* 0.05). The 3D contour plot of the effects of Span® 60 and cholesterol concentrations on EE is shown in Figure 2C.

#### 3.1.5 Content Release

The percentage of release of niosome formulations was between 65.37 and 34.38% (Supplementary Table S2). According to results for release after 24 h, the best-fitting model for release was quadratic and statistically significant (*p =* 0.026; Supplementary Table S3). Regression analysis of the percentage of release based on the quadratic model (Supplementary Table S4) indicates that both Span^®^ 60 and cholesterol have a positive effect on release. The 3D contour plot of the effects of Span® 60 and cholesterol on release is shown in Figure 2D.

#### 3.1.6 Validity of the Central Composite Design (CCD)

The CCD design is considered valid when the values of adjusted R^2^ and the predicted R^2^ are within 0.20 (Moghtaderi et al., 2021). According to the regression data for various responses (Supplementary Table S5), there is a good agreement between R^2^ and adjusted R^2^.

#### 3.1.7 Data Optimization

**FIGURE 3:**
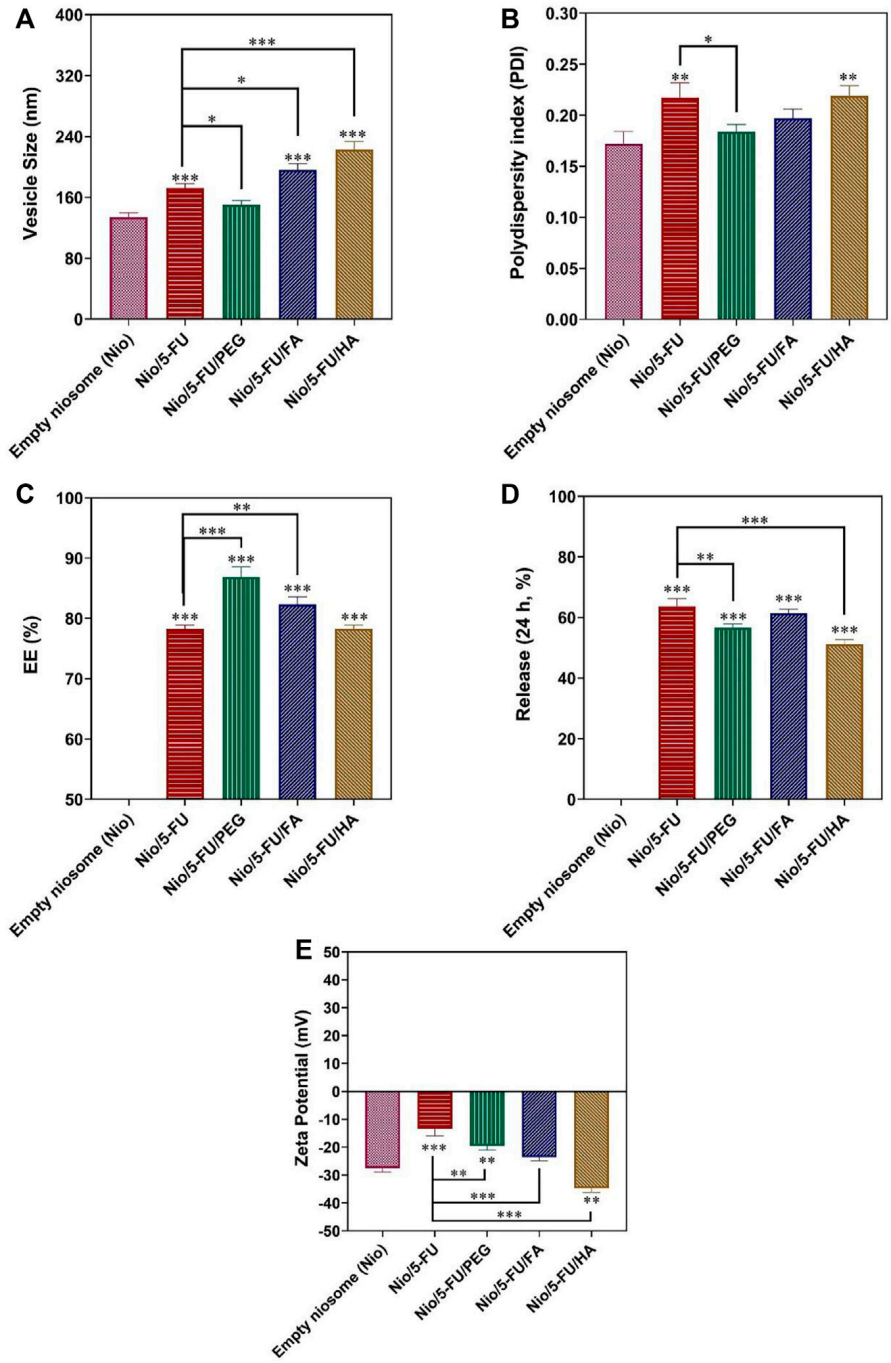
Optimized responses were obtained by coated and uncoated formulations under the optimal conditions. **(A)** Vesicle size. **(B)** Polydispersity. **(C)** Entrapment efficacy. **(D)** Release percentage. **(E)** Zeta Potential. Data represent means ± standard deviations (*n* = 3). For all charts, ***: *p <* 0.001; **: *p <* 0.01; *: *p <* 0.05.

The design of a suitable nanocarrier was carried out after predicting the optimal particle size, PDI, and EE% by the CCD method. A good desirability index (desirability = 0.833) was achieved with a cholesterol: Span^®^ 60 M ratio of 1.66 (i.e., cholesterol: 3.082 mM; Span^®^ 60: 1.852 mM). This index is a multi-criteria optimization algorithm used when one response must be at the minimum, and the other must be at the maximum to achieve the optimal formulation. The optimal condition for narrow particle size, minimum polydispersity, maximum entrapment efficacy, and proper release percentage was predicted (Figure 3). Optimization was valid because its desirability index was 0.83. The particle size, polydispersity index, entrapment efficacy, and release of the predicted nanocarriers were 194.795 nm, 0.234, 79.92 %, and 54.39%, respectively. According to Figure 3, the validity of the CCD design was clear because there was no significant difference between experimental data for the 5-FU loaded pristine niosomes and the decorated niosomes. The optimized 5-FU-loaded, PEG-coated niosomes (Nio/5-FU/PEG) had smaller diameters (150.4 nm), smaller PDI (0.18), higher drug entrapment (86.91 %), and lower drug release (Figure 3D) than the undecorated optimized formulation (Nio/5-FU). The formulations Nio/5-FU and Nio/5-FU/PEG had PDI values of 0.2 and 0.18, respectively. The PDI value of coated Nio/5-FU was narrower than the non-coated Nio/5-FU because Nio/5-FU/PEG had a smaller size distribution and was a more homogeneous system. Compared to undecorated niosomes, the Nio/5-FU/PEG showed a better EE% (86.9%). Polyethylene glycol is hydrophilic, and this property renders the surface of the niosomes hydrophilic. As a result, both the vesicle size and the EE% increased. According to Figure 3C, the optimized 5-FU-located, folic acid-decorated niosomes (Nio/5-FU/FA) had higher drug entrapment property (82.35 %), larger diameter (196.3 nm), lower PDI value (0.19), and a lower drug release profile than the optimized pristine niosome formulation Nio/5-FU. The EE% of Nio/5-FU and Nio/5-FU/FA were 78 and 82.3%, respectively (Figure 3C). The value of EE% is dependent on the particle size. It has been reported that the fluidity of the bilayer affects the stability of niosomes (Junyaprasert et al., 2013). Accordingly, decorating the niosomes with FA increases both particle size and EE%. The increase in PDI value prevented aggregation of niosomes due to electrostatic stabilization (Mahale et al., 2012). After coating the pristine Nio/5-FU with HA, the particle size increased from 194.7 to 223 nm (Figure 3A), and the PDI value changed from 0.217 to 0.219 (Figure 3B).

### 3.2 Zeta Potential

Zeta Potential is attributed to the stability of the carrier (Manconi et al., 2017). The Zeta Potential graph is shown in Figure 3E. All niosome formulations have a negative Zeta Potential, which shows that they have good stability due to the weak electrostatic repulsive force in the niosomal bilayer. The zeta value of empty Nio was −27.54 ± 1.34 mV, and the Zeta Potential of Nio/5-FU was −13.45 ± 2.55. After HA-coated (Nio/5-FU/HA), the Zeta Potential changed from −13.45 ± 2.55 mV to −34.73 ± 1.50 mV. Nio/5-FU/PEG obtained in this experiment had a Zeta Potential of −19.54 ± 1.49. As seen in Figure 3E, Nio/5-FU/FA had a negative Zeta Potential (−23.64 ± 1.22) than Nio/5-FU.

### 3.3 Size Distribution and Morphology of the Nanoformulations

**FIGURE 4:**
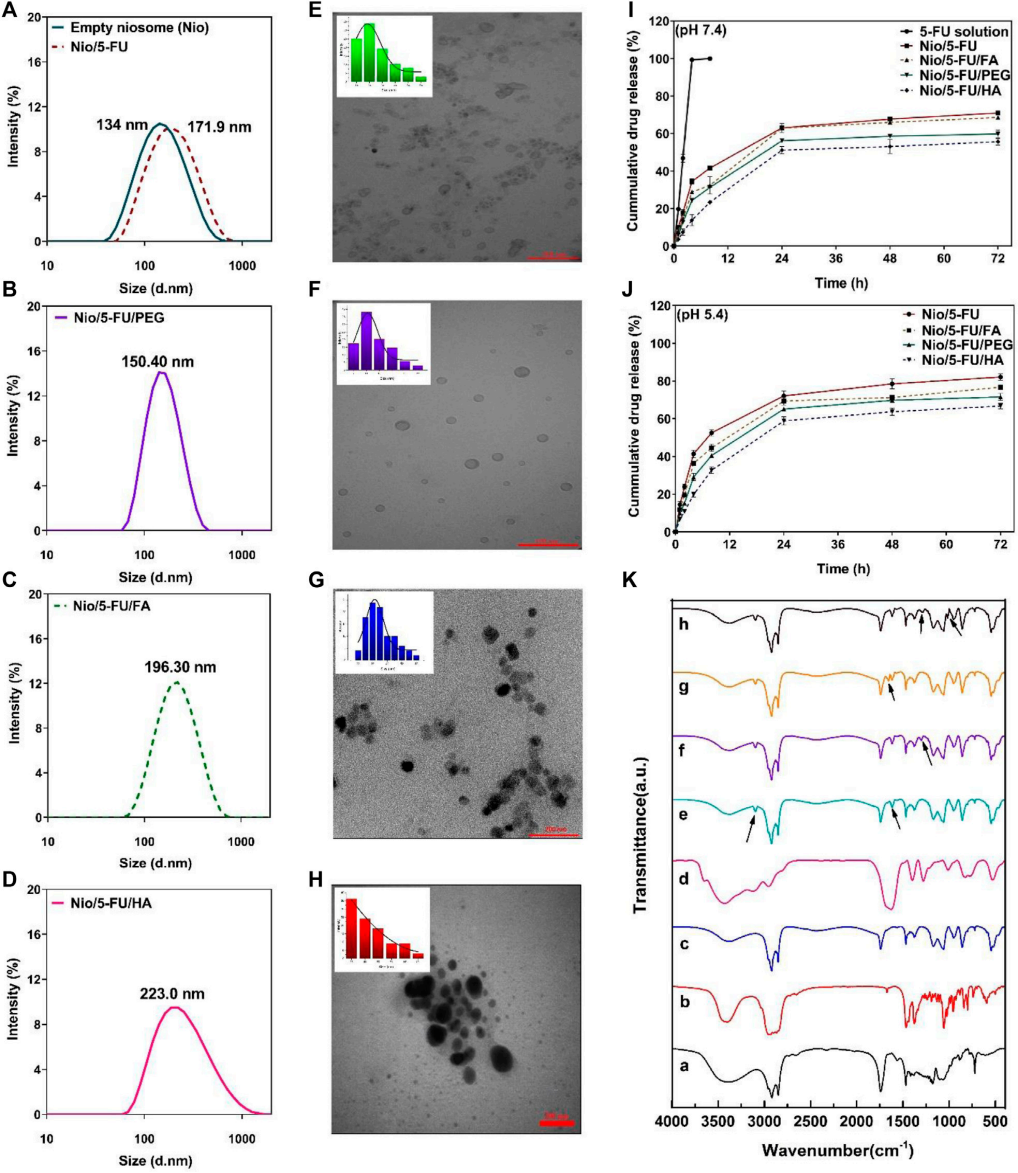
**(A–D)** The particle size of synthesized niosomes as determined by dynamic light scattering. **(E)** Transmission electron micrograph and size distribution of Nio/5-FU. **(F)** Transmission electron micrograph and size distribution of Nio/5-FU/PEG. **(G)** Transmission electron micrograph and size distribution of Nio/5-FU/FA. **(H)** Transmission electron micrograph and size distribution of Nio/5-FU/HA. **(I)** *In vitro* release of 5-FU from different niosomal formulations at pH 7.4: 5-FU-loaded pristine niosomes (Nio/5-FU) and those that had been decorated with folic acid (FA), polyethylene glycol (PEG) or hyaluronic acid (HA). (Nio/5-FU/FA). **(J)** *In vitro* release of 5-UL from different niosomal formulations at pH 5.4. **(K)** Fourier transform infrared FTIR spectra of **a**) Span 60, **b**) Cholesterol, **c**) niosome, **d**) 5-FU, **e**) Nio/5-FU, **f**) Nio/5-FU/PEG, **g**) Nio/5-FU/HA, and **h**) Nio/5-FU/FA.

The morphology of niosome-based carriers was examined by transmission electron microscopy. The size distribution of undecorated and decorated Nio/5-FU formulations was determined by dynamic light scattering (DLS) with a particle size analyzer (Figures 4A–D). The particle size of synthesized niosomes was measured using ImageJ software (National Institute of Health, Bethesda, MD, United States). According to the morphology of prepared niosomes formulations, it can be seen from transmission electron micrograph (TEM) that all niosomes were spherical and homogeneous. Furthermore, no drug crystal was observed in any of the images (Figures 4E–H). Since the morphology of prepared noisome formulations showed that Nio’s vesicle scales were less than 100 nm, all formulations have excellent size for cancer applications. Among coated and uncoated niosomal formulations, Nio-5-FU-PEG (Figure 4F) sample shows more homogeneous and spherical than Nio-5-FU-FA (Figure 4G) and Nio-5-FU-HA (Figure 4H). Also, this niosomes coated with PEG have the lowest average particle size (15.14 nm) in compared with Nio-5-FU-FA and Nio-5-FU-HA. Furthermore, the aggregates in Nio/5-FU/PEG (Figure 4F) were less than other noisome formulations, which shows that the coated formulation with PEG is more stable. The size of the noisome loaded with FU was wider when the niosomes were coated with HA (55.9 nm) (Figure 4H) in compared to two other niosomal formulations. Furthermore, the diameters of the vesicles increased with FA addition compared to uncoated niosomes 40.6 nm (Figure 4G) and 34.9 nm (Figure 4E), respectively. A possible reason for this result is that adding FAPEG2000 causes an increase in the surface tension of the membrane bilayer and higher surface tension helps to decrease the fluidity of the bilayer and increase the particle size of niosomes (Liu and Guo, 2005).

### 3.4 Drug Release

Release of 5-FU from the niosomes was measured for 72 h in phosphate-buffered saline-sodium dodecyl sulfate at physiological pH (7.4) and pathological (cancerous) pH (∼5.4). There was an initial burst release of 5-FU during the first 2 h. The release rates of 5-FU from Nio, Nio/FA, Nio/HA, and Nio/PEG in physiological pH (Figure 4I) were significantly lower than that of undecorated and decorated niosomes at pathological (cancerous) pH (Figure 4J). It is because of the typical behavior of the niosomes at acidic condition, which result in swelling and breaking niosome in this condition. Furthermore the reason for this can be attributed to the electrostatic interaction between the drug and the surfactant, and there is an ionization state at physiological pH (Dash et al., 2010b). After the initial burst release attributed to diffusion of 5-FU from the outer layer of the niosomes, all formulations exhibited the second phase of slower release up to 72 h. The decorated niosomes Nio/5-FU/FA, Nio/5-FU/HA and Nio/5-FU/PEG had lower release rates than the uncoated niosomes. The release rate of Nio/5-FU/HA was relatively lower compared to other undecorated and decorated formulations.

### 3.5 Modeling of Release Kinetics

The different release kinetics models obtained for optimizing niosomal formulations are shown in Supplementary Table S6. The best-fitting model for 5-FU, as determined by the highest R^2^ value for curve-fitting, was the first-order model because the value R^2^ for the first-order model in 5-FU was 0.9695. In this model, there is a correlation between drug release and concentration. The best model for optimized Nio/5-FU, in two different pH scenarios, was the Higuchi, because its value was 0.9649 and 0.9754, respectively. The best-fitting model for Nio/5-FU/PEG, and Nio/5-FU/FA in two different pH scenarios was the Higuchi, drug release model (Nio/5-FU/PEG: 0.9340 at pH 7.4 and 0.9408 at pH 5.4, Nio/5-FU/FA: 0.9452 at pH 7.4 and 0.9571 at pH 5.4). In contrast, the best model for Nio/5-FU/HA was the Korsmeyer-Peppas model. the value R^2^ for the Korsmeyer-Peppas model in Nio/5-FU/HA in pH 7.4 and pH 5.4 was 0.9470 and 0.9529, respectively. Nio/5-FU/PEG, and Nio/5-FU/FA in two different pH scenarios was the Higuchi, drug release model because. The classical Higuchi equation is represented by: Q = A [D (2Co-Cs)Cst]½, where Q is the cumulative amount of drug released in time t per unit area, CO is the initial drug concentration, CS is the drug solubility in the matrix and D is the diffusion coefficient of the drug molecule in the matrix.

### 3.6 FTIR Spectroscopy

The empty noisome exhibits stretching peaks for C-O, C=O, and C-H at 1125, 1747, and 2900 cm^−1^, respectively. Additionally, it exhibits a carbonyl bond at 1625 cm^−1^ and a -NH stretching vibration at 3100–3400 cm^−1^, indicating that noisome was successfully formed. Following drug loading, the carbonyl group shifted to 1614 cm^−1^ and the stretching amide group to 3095 cm^−1^, indicating the presence of 5-fu in the niosome structure. Following PEGylation of the structure of the drug-loaded niosome, the C-H group corresponds to the PEG structure visible in the final formulation’s 1300 cm^−1^ region, confirming the structure’s PEGylation. After adding hyaluronic acid to the drug-loaded niosome, a peak at 1655 cm^−1^ corresponding to the amide group appears in the region, confirming the successful incorporation of HA into the final structure. The final stage of the work involved adding folic acid to the final structure (drug-loaded niosome), which resulted in the appearance of the C-N stretching group in the 1015 cm^−1^ and 1279 cm^−1^ regions, confirming the presence of folic acid in the final formulation (Figure 4K).

### 3.7 Stability

The stability of undecorated and decorated niosomes and the percentage of residual 5-FU, was examined after storage at 4 and 25°C for 30 days. The Nio/5-FU/PEG nanocarriers were more stable than the other niosomes. Significant differences in PDI and EE were observed for the Nio/5-FU and Nio/5-FU/PEG formulations after storage at 4 and 25°C. The increase in size and PDI and the decrease in EE at 4°C were lower than 25°C after storage (Supplementary Table S8). Although the Nio/5-FU/PEG niosomes were physically stable during storage, leakage of 5-FU occurred for the other niosomal formulations. A more severe leakage was observed for these formulations when stored at 25°C. Changes in particle size and PDI were also more prominent at 25°C. This is because the niosomal nanocarriers have lower mobility and permeability at 4°C. These results suggest that the Nio/5-FU/PEG niosomes are more stable than other niosomes and are more stable at 4°C than 25°C. Supplementary Table S7 indicates that similar to other niosomal formulations, the size, PDI and the EE% of Nio/5-FU/FA increased with increased storage temperature. However, the EE of Nio/5-FU/FA was more sensitive to temperature when compared with the other niosomal formulations. The Nio/5-FU/FA could be stored at 25°C for up to 1 month with only minor changes in size and drug content.

### 3.8 Cell Proliferation

**FIGURE 5:**
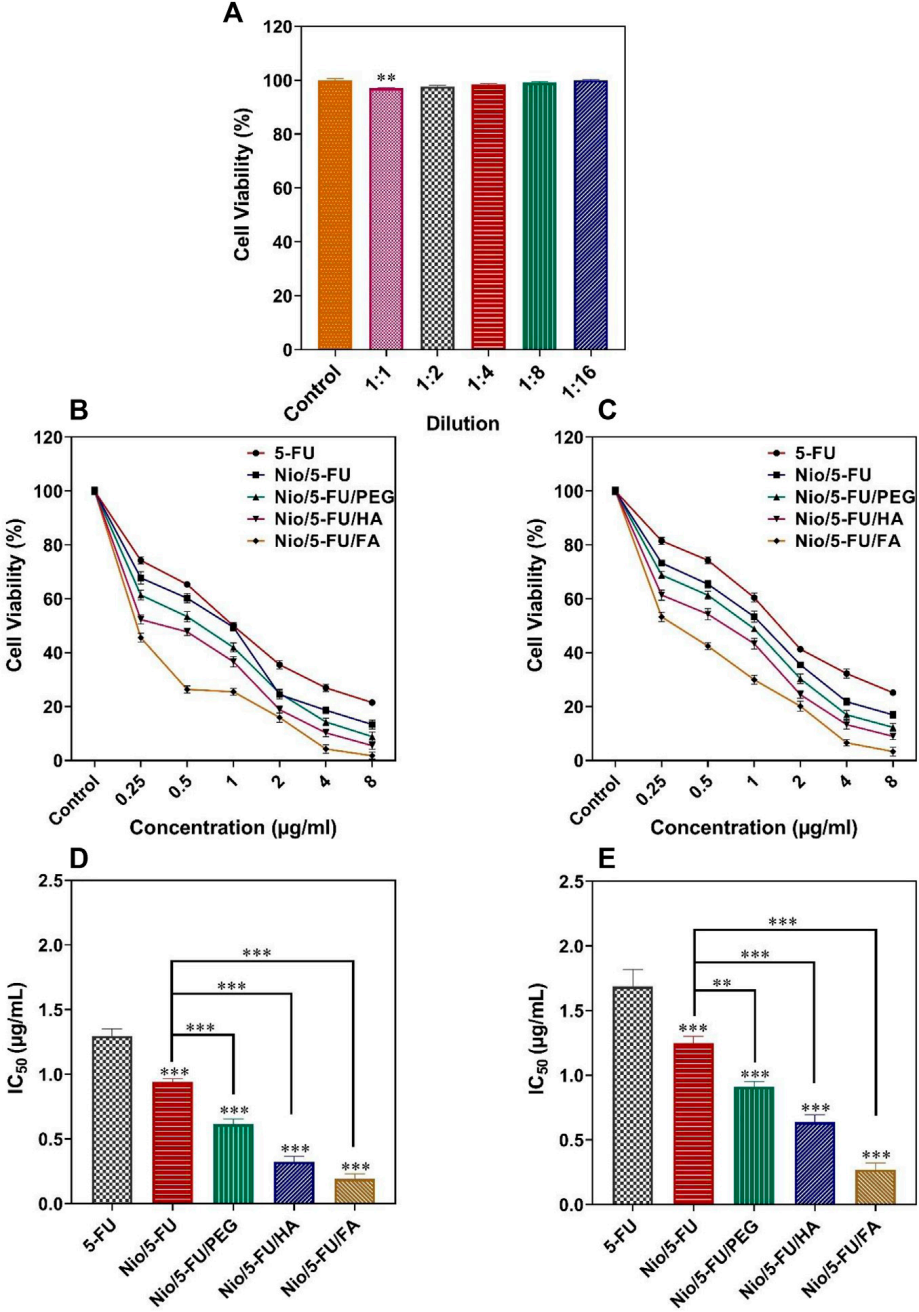
**(A)** Percentage cell viability of different dilutions of niosomes on non-malignant MCF10A cells. **(B)** The effects of 5-FU, Nio/5-FU, Nio/5-FU/PEG, Nio/5-FU/HA and Nio/5-FU/FA on the viability of MCF7 cells. **(C)** The effects of 5-FU, Nio/5-FU, Nio/5-FU/PEG, Nio/5-FU/HA and Nio/5-FU/FA on the viability of 4T1 cells. **(D)** Half-maximum inhibitory concentration (IC_50_) values after 48 h of exposure of MCF7 breast cancer cells to 5-FU, Nio/5-FU, Nio/5-FU/PEG, Nio/5-FU/HA and Nio/5-FU/FA. **(E)** IC_50_ values after 48 h treatment of malignant 4T1 cells to 5-FU, Nio/5-FU, Nio/5-FU/PEG, Nio/5-FU/HA and Nio/5-FU/FA. Data represent means ± standard deviations (*n* = 3). For all charts, ***: *p <* 0.001; **: *p <* 0.01; *: *p <* 0.05.

Apart from the 1:1 dilution, there were no statistically significant changes in the viability of MCF10A cells (non-malignant breast epithelial cells) after they were exposed to different concentrations of niosomes obtained by serial dilutions (*p* < 0.05; Figure 5A). The cell toxicity results for each formulation at different times are reported in Figures 5B,C. The effect of the different niosome formulations on the survival of MCF7 breast cancer cells is summarized in Figure 5D. The half-maximal inhibitory concentration (IC50) values of the different formulations were: 5-FU (1.29 ± 0.06 μg/ml), Nio/5-FU (0.94 ± 0.03 μg/ml), Nio/5-FU/PEG (0.61 ± 0.04 μg/ml), Nio/5-FU/HA (0.32 ± 0.04 μg/ml) and Nio/5-FU/FA (0.19 ± 0.03 μg/ml). Compared to 5-FU, there were also significant decreases in the IC50 values of Nio/5-FU, Nio/5-FU/PEG, Nio/5-FU/HA and Nio/5-FU/FA (*p* < 0.001, *p* < 0.001, *p* < 0.001, and *p* < 0.001, respectively). The Nio/5-FU/PEG, Nio/5-FU/HA and Nio/5-FU/FA also showed statistically significant decreases in IC50 compared with Nio/5-FU (*p* < 0.001).

The effect of the niosome groups on the survival of malignant 4T1 cells (a breast cancer cell line derived from the mammary gland of a BALB/c mouse) is summarized in Figure 5E. The IC50 values for the different formulations were 5-FU (1.69 ± 0.13 μg/ml), Nio/5-FU (0.94 ± 0.03 μg/ml), Nio/5-FU/PEG (0.91 ± 0.04 μg/ml), Nio/5-FU/HA (0.64 ± 0.05 μg/ml) and Nio/5-FU/FA (0.27 ± 0.05 μg/ml). Compared to 5-FU, there were significant decreases in the IC50 values of Nio/5-FU, Nio/5-FU/PEG, Nio/5-FU/HA and Nio/5-FU/FA (*p* < 0.001 for all comparisons). The IC50 values for Nio/5-FU/PEG, Nio/5-FU/HA and Nio/5-FU/FA were significantly lower than those of Nio/5-FU (*p* < 0.01, *p* < 0.001, and *p* < 0.001, respectively). There is an interact between niosomal formulation and folate receptors on the surface of these breast cancer cell lines. As a result, a folate receptor-mediated endocytosis can enhance the internalization process of folic acid functionalized niosomes. This was further conformed by comparing Fu loaded to niosomal formulation with FA and other niosomal modifications (Nio/5-FU/PEG and Nio/5-FU/HA) where the functionalized niosomal formulation with folic acid resulted in greater toxicity in cancer cells after 48 h of treatment compared to two other niosomal (Akbarzadeh et al., 2020a). Moreover, Nio/5-FU/FA exhibited a significant increase in cell inhibition activity when compared with Nio/5-FU/PEG and Nio/5-FU/HA. It is possible that Nio/5-FU/FA further the uptake into cell in turn enhanced cellular toxicity. This study approved earlier results, which showed that FA-modified formulations exhibited the greatest cytotoxicity as a result more effective inhibited cell growth (Lei et al., 2017; Lin et al., 2020).‏

### 3.9 Flow Cytometry

**FIGURE 6:**
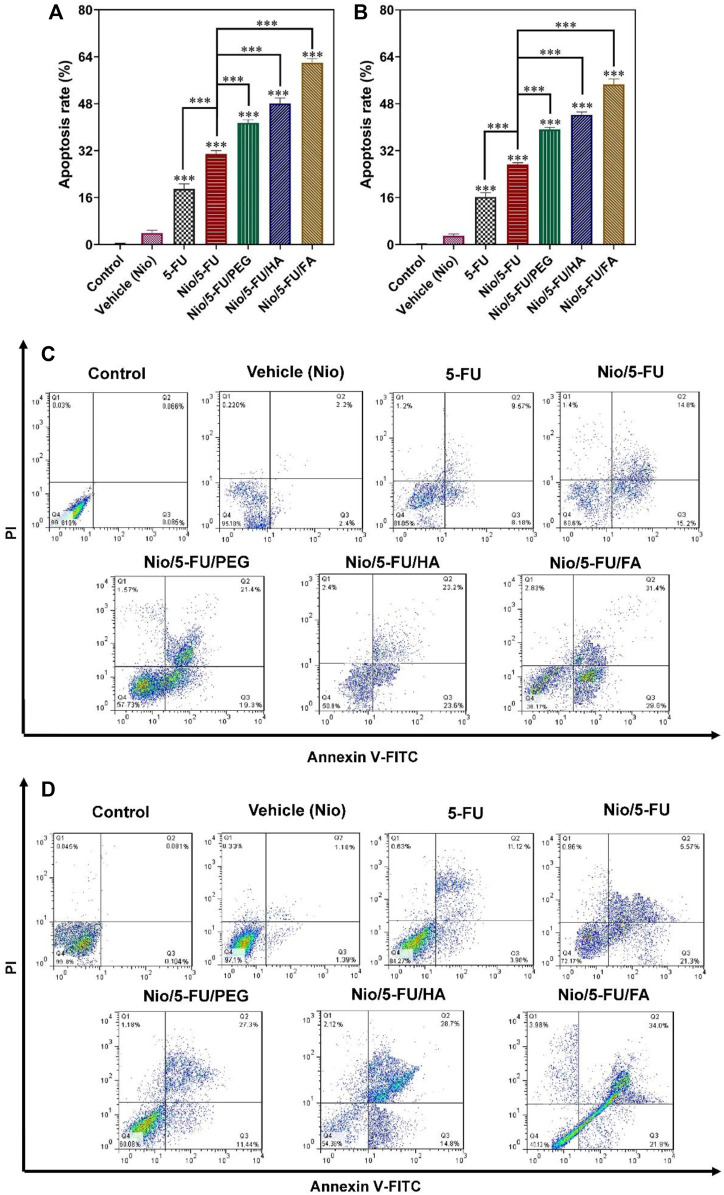
The effects of control, 5-FU and different niosome formulations (Nio), 5-FU, Nio/5-FU, Nio/5-FU/PEG, Nio/5-FU/HA and Nio/5-FU/FA of **(A)** MCF7 and **(B)** 4T1 breast cancer cells that became apoptotic after 48 h of treatment. Data represent means ± standard deviations (*n* = 3). For all charts, ***: *p <* 0.001; **: *p <* 0.01; *: *p <* 0.05. **(C,D)** Flow cytometric analysis of (C) MCF7 and (D) 4T1 cells after treatment with IC50 concentration of vehicle (Nio), 5-FU, Nio/5-FU, Nio/5-FU/PEG, Nio/5-FU/HA and Nio/5-FU/FA formulations. The upper left square (Q1) shows the percentage of necrotic cells, and the upper right square (Q2) exhibits the percentage of late apoptotic cells, (Q3) exhibits the percentage of early apoptotic cells, and (Q4) shows the percentage of live cells.

Apoptosis of breast cancer cells was measured quantitatively with flow cytometry after the cells were double-stained with fluorescein thiocyanate-labeled annexin V (annexin V-FITC) and propidium iodide (PI). Administration of Nio, 5-FU, Nio/5-FU, Nio/5-FU/PEG, Nio/5-FU/HA, and Nio/5-FU/FA to the MCF7 cells (Figures 6A,C) and the 4T1 cells (Figures 6B,D) induced apoptosis of both types of breast cancer cells. The percentages of apoptotic cells in both cell lines treated with Nio were not significantly different from the control cells that were not exposed to any of the niosomal formulations (*p >* 0.05). There were significant increases in the percentage of apoptotic MCF7 cells over the control after the breast cancer cells were exposed to 5-FU, Nio/5-FU, Nio/5-FU/PEG, Nio/5-FU/HA and Nio/5-FU/FA (*p <* 0.001). The percentages of apoptotic MCF7 cells after treatment with Nio/5-FU/PEG, Nio/5-FU/HA and Nio/5-FU/FA increased significantly, compared to those treated with Nio/5-FU (*p <* 0.001; Figure 6A). On the contrary, the percentage of apoptotic cells decreased when 5-FU was used instead of the undecorated niosome nanocarriers (*p <* 0.001). Similar trends were identified for the 4T1 murine breast cancer cells (Figure 6B). Pairwise comparisons indicated that the anticancer effect of Nio/5-FU/FA on the two cell types was higher than Nio/5-FU/PEG or Nio/5-FU/HA.

### 3.10 ROS Assay

**FIGURE 7:**
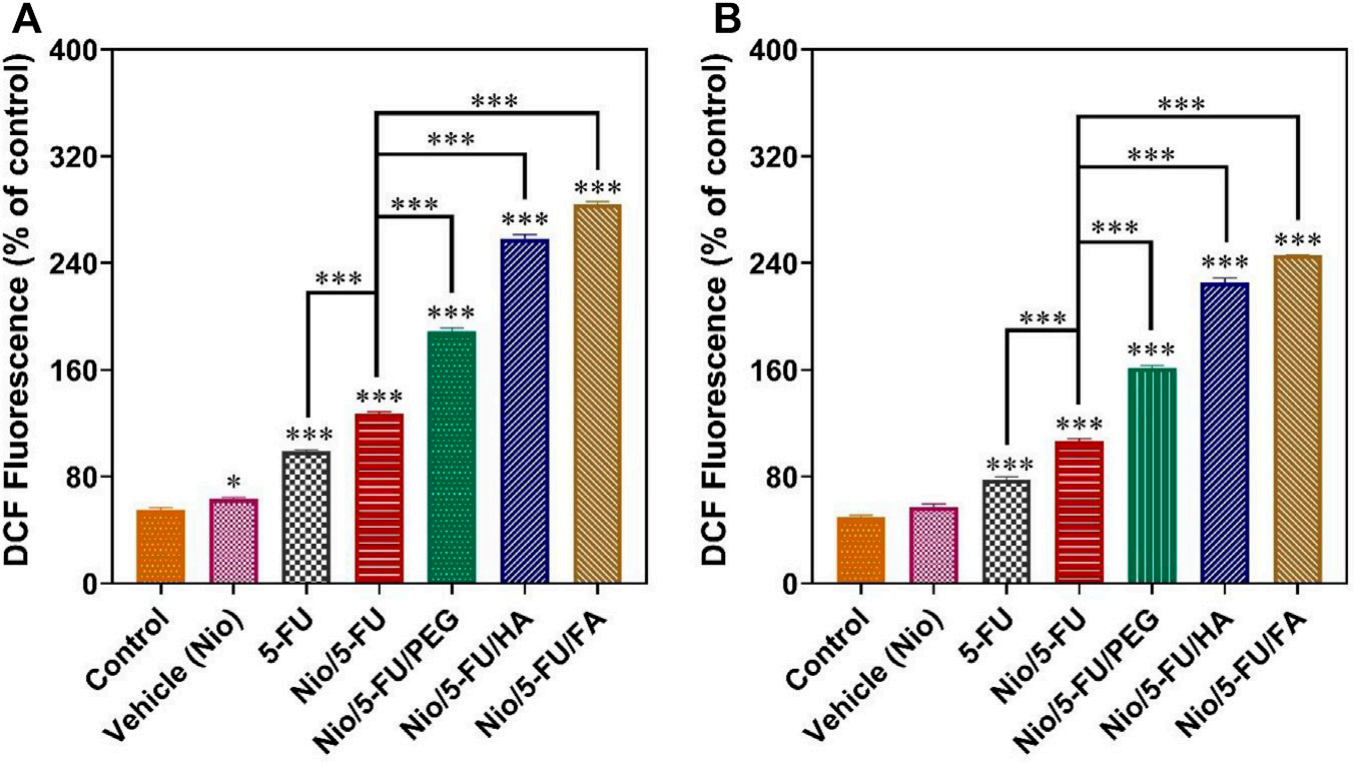
Changes in intracellular ROS content, indicated by the fluorescence of 2′, 7′-dichlorodihydrofluorescein (DCF), are summarized in **(A)** for MCF7 cells and **(B)** for 4T1 cells. Data represent means ± standard deviations (*n* = 3). For all charts, ***: *p <* 0.001; **: *p <* 0.01; *: *p <* 0.05.

An important causative factor for cell death of tumor cells is oxidative stress (Joo and Jetten, 2010). Although a small increase in the level of reactive oxygen species (ROS) helps promote cell growth, extreme levels of ROS stimulate cell death. Compared to the control, 5-FU, Nio, Nio/5-FU, Nio/5-FU/PEG, Nio/5-FU/HA, and Nio/5-FU/FA enhanced the fluorescence of 2′,7′-dichlorodihydrofluorescein (DCF), a fluorescent probe for measuring ROS, in both MCF7 cells (Figure 7A) and 4T1 cells (Figure 7B). The 4T1 cells exposed to Nio did not show any significant changes compared to the control group. In contrast, the MCF7 cells exposed to 5-FU, Nio/5-FU, Nio/5-FU/PEG, Nio/5-FU/HA and Nio/5-FU/FA showed significant increases in DCF fluorescence compared to the control (*p <* 0.001). DCF fluorescence of MCF7 human breast cancer cells treated with decorated niosomes was significantly increased compared to cells exposed to Nio/5-FU (*p <* 0.001) (Figure 7A). On the contrary, a significant reduction in fluorescence occurred for cells exposed to 5-FU (*p <* 0.001). Similar results were observed with the 4T1 murine breast cancer cells (Figure 7B).

### 3.11 Confocal Laser Scanning Microscopy

**FIGURE 8:**
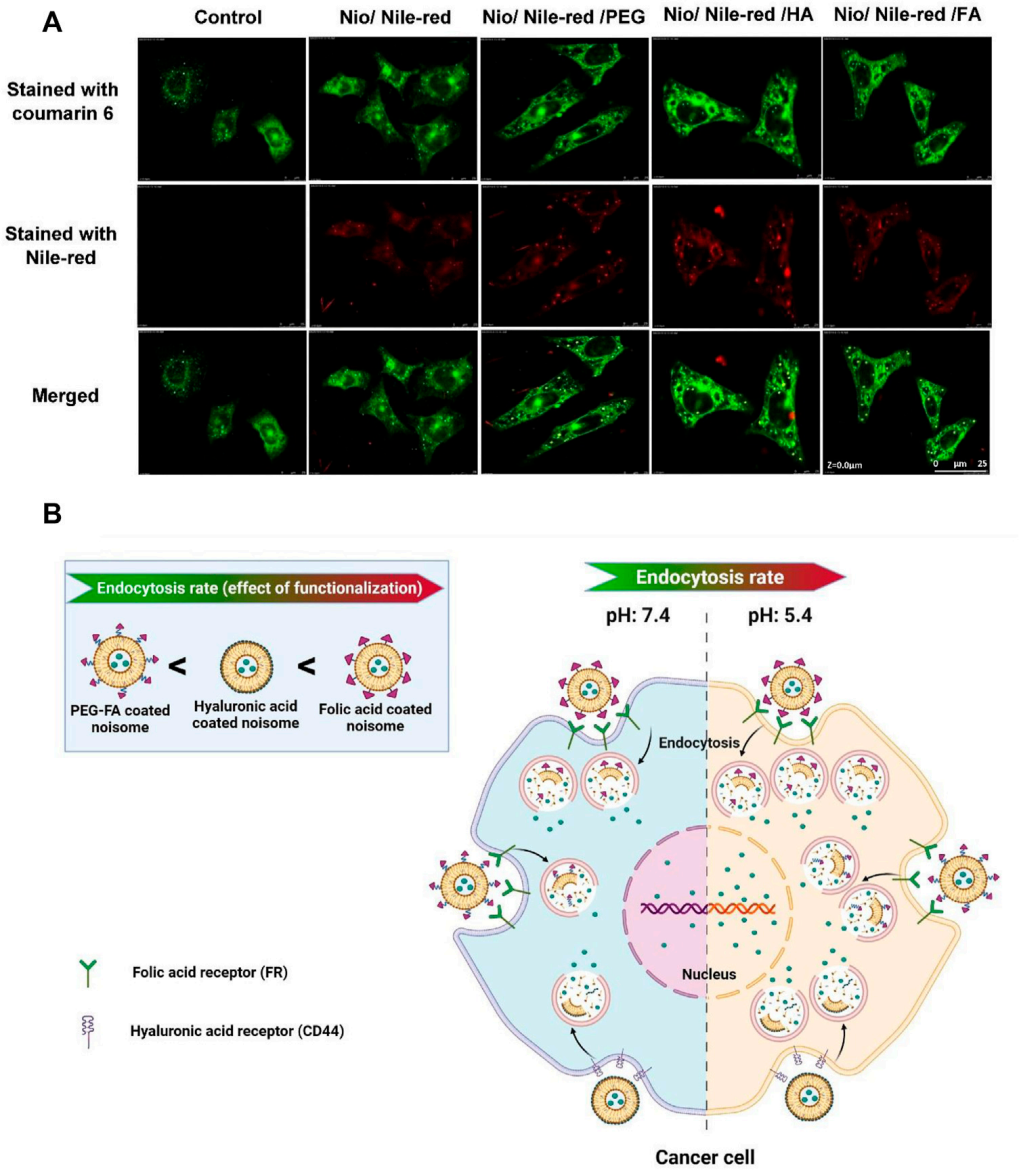
The uptake of niosomes in MCF7 cells was investigated with confocal laser scanning microscopy. The niosomes investigated were: Nio/5-FU, Nio/5-FU/PEG, Nio/5-FU/HA and Nio/5-FU/FA. **(A)** Representative CLSM images of MCF7 cells stained with coumarin 6 and Nile-red. **(B)** Schematic depicting the effect of pH on the release of contents from a niosome.

Confocal laser scanning microscopy (CLSM) was used to study the uptake of niosomes into cancer cells. Nile red was used as a hydrophobic model molecule. As shown in Figure 8A, Nile red-containing niosomes were readily uptaken by MCF7 cells and localized in the cytoplasm. Compared with cells exposed to 5-FU loaded pristine niosomes (Nio/5-FU) and the control cells that were not exposed to anything niosome formulation, there was more profuse penetration of the 5-FU-located, decorated niosomes into the cancerous cells. As expected, the targeted niosomes were beneficial in promoting cellular uptake in comparison with free 5-FU. In the case of Nio/5-FU/HA and Nio/5-FU/PEG, Nile red was significantly localized probably outside the cells instead of a distribution in the endosomes, indicating that the Nio/5-FU/PEG HA and Nio/5-FU/PEG had less cell uptake than Nio/5-FU/FA. The Nio/5-FU/FA shows a significant profuse uptake and better anticancer effect compared to the free 5-FU drugs, Nio/5-FU/HA and Nio/5-FU/PEG. The differences were attributed to the chemical reaction between the folate drug-loaded conjugates and the folate receptor cells in the membrane of cancer cells, which may be explained by the fact that cancer cells overexpress the folate receptor since folic acid is a high-affinity ligand for the folate receptor. As a result, modified niosomes with FA facilitated efficient cellular uptake of the Nio/5-FU/FA into the tumor cells leading to better cytotoxicity in the process than the free 5-FU anticancer drugs, Nio/5-FU/HA and Nio/5-FU/PEG (Lin et al., 2020). The release rate of 5-FU from the FA, HA or PEG-decorated niosomes at pH 5.4 was higher than the free drug and the release rate at pH 7.4. In addition, endocytosis was the most prominent in the cells exposed to Nio/5-FU/FA, compared with cells exposed to Nio/5-FU/HA or Nio/5-FU/PEG (Figure 8B). The variation of the pH in the endosome cause to dissociation of folate from the receptors, which can be recycled back to the membrane. As a result, Nio/5-FU/FA efficiently releases drugs into cancer cells compared to Nio/5-FU/HA and Nio/5-FU/PEG (Tavano et al., 2016).

## 4 Discussion

Nanomedicine increases the effectiveness of breast cancer treatment and reduces the associated side effects (Gulla et al., 2021; Hu et al., 2021; Wen et al., 2021). Surface modifiers such as PEG, FA, and HA have been employed to facilitate the accumulation of nanocarriers in tumor cells. The results demonstrated that the particle size of the synthesized niosomes increased with the higher cholesterol to Span^®^ 60 M ratio. This result might be for the higher amount of 5-FU enclosed inside the vesicles. Furthermore the increase in particle size with increasing Span 60 concentration was also observed by Zaki et al. working on diacerein-loaded niosomes (Naresh et al., 1994; Liu and Guo, 2005). At the low concentrations of cholesterol, the vesicular membranes are more flexible and more liable to be affected by ultrasound waves, resulting in smaller niosomes. An overall increase in the cholesterol concentration, rigidity of the vesicular membranes, and the resistance post-sonication leads to the generation of larger-sized particles (Essa, 2014; Qumbar et al., 2017). On the other hand, as shown in the literature, increasing Span^®^ 60 concentration results in a less permeable niosomal membrane, which increases EE (Kaushik et al., 2006). Functionalization of niosomes with PEG leads to the production of a less but larger bilayer of lipids, which causes growth in the particle size of pure niosomes. This is because of the hydrophilic properties of PEG (Gabizon et al., 2012). PEG-coated nanoformulations showed less aggregation, smallest size, and highest entrapment efficiency compared to other functionalized niosomes (e.g., FA and HA). The diameter of 5-FU/Nio/FA was also larger than that of the pure niosome, as FA-PEG2000 increases the surface tension of the bilayer membrane, followed by a reduction in the fluidity of the bilayer. However, the polydispersity index of Nio/5-FU/FA was reduced compared to the pristine niosome due to increasing the electrostatic repulsion after surface modification with FA (Lin et al., 2020). The Nio/5-FU/HA nanocarriers had the largest amount of Zeta Potential among all samples as the anionic character of HA has a significant effect on Zeta Potential and electrostatic stabilization (Bayindir and Yuksel, 2010; Martens et al., 2015). The morphology result indicated that Nio/5-FU/PEG nanoparticles were more homogeneous and spherical with a smaller size than other samples. In addition, the aggregates in Nio/5-FU/PEG were less than other niosome formulations, showing that the coated nanoniosomes with PEG are more stable. The particle size, PDI, and entrapment efficiency changes between two temperatures (4 and 25°C) showed that Nio/5-FU/PEG formulation was more stable than the other coated and uncoated niosomes. Stability showed that PEGylating niosomal formulations has an important role in minimizing problems related to niosomal instability, such as aggregation, fusion, and drug leakage (Naderinezhad et al., 2017).

The drug release study showed a pH-dependent profile due to the electrostatic interaction between the drug and the surfactant and ionization state at physiological pH (Korsmeyer et al., 1983; Dash et al., 2010a). 5-FU release rates from uncoated Nio, Nio/FA, and Nio/HA were lower at physiological pH (neutral environment) than pathological cancerous pH (acidic environment). Besides, the decorated niosomes showed a lower release rate than pristine niosomes in both conditions.

In the present study, the cellular and molecular effects of 5-FU loaded into FA, HA, or PEG-decorated niosomes against MCF7 and 4T1 breast cancer cells. 5-FU is an anti-metabolite drug of the pyrimidine analog group that can interfere with nucleoside metabolism, is embedded in RNA and DNA, and leads to cytotoxicity and death in tumor cells (Arias, 2008). Endocytosis was enhanced in 5-FU loaded into FA-targeted niosome compared to 5-FU loaded into HA and PEG-targeted niosome. In addition, drug-free niosomes had no toxic effects on healthy cells.

It is well-known that extreme levels of ROS cause cell death. ROS continuously produced in biological systems and cause damage to DNA, proteins, and lipids (Tomar et al., 2019). High level of ROS even modifies the function of proteins through the regulation of redox-sensitive proteins, gene expression, redox receptor-binding proteins, redox-sensitive enzyme-modifying enzymes, and protein turnover regulation (Joo and Jetten, 2010; Sadri et al., 2020). Cancer cells produce more ROS than normal cells due to hypoxia, mutations in nuclear and mitochondrial genes, oncogenes activation, and tumor suppressor genes loss. In cancer cells, low to moderate levels of ROS are essential for cell development, differentiation, and survival, but at high levels it leads to cell death. Recent evidence suggests the role of ROS as a messenger in tumor cell invasion, angiogenesis, and metastasis (Zeng et al., 2016). In this study, the amount of ROS in cancer cells treated with Nio/5-FU/FA showed significant growth compared to other groups.

## 5 Conclusion

In the present study, 5-FU/Nio was coated with different biomolecules, including PEG, HA, and FA. There was a significant difference in size, PDI and EE% between uncoated Nio/5-FU and coated Nio/5-FU formulations, which were stored at both 4 and 25°C. All targeted niosome formulations (Nio/5-FU/PEG, Nio/5-FU/FA and Nio/5-FU/HA) had higher entrapment efficiency than uncoated niosomes. The Nio/5-FU/PEG had higher drug entrapment in comparison with Nio/5-FU/FA and Nio/5-FU/HA because of the hydrophilic property of Polyethylene glycol. The result indicated that functionalized nano niosomes were more stable. The nanocarriers showed high breast cancer cell sustained release at pH = 7.4, while faster release in acidic pH (∼5.5) was observed. This study manifests that Nio/5-FU/FA-based nanocarriers possess suitable physicochemical properties and a high encapsulation efficacy. Also, Nio/5-FU/FA compared to Nio/5-FU/PEG and Nio/5-FU/HA *via* increased MCF7 and 4T1 breast cancer cell cytotoxicity, and an increased amount of ROS could lead cancer cells to apoptosis. All in all, the proposed nanocarriers can be promising systems for the treatment of breast cancers.

## Data Availability

The original contributions presented in the study are included in the article/Supplementary Material, further inquiries can be directed to the corresponding authors.
