# Supplementary material for: Folic Acid-Decorated pH-Responsive Nanoniosomes With Enhanced Endocytosis for Breast Cancer Therapy: In Vitro Studies
Source: Front Pharmacol. 2022 Apr 20;13:851242. doi: 10.3389/fphar.2022.851242 (PMC9065559; doi:10.3389/fphar.2022.851242)
Supplement: Supplementary file 1 [file DataSheet1.PDF]

Supplementary Material

Table S1. Different levels for variables in the Central Composite Design (CCD) optimization. Drug concentration: 1 mg/mL; Sonication time: 5 min.

Level	-1	0	+1
A (Span® 60, mM)	2	4	6
B (Cholesterol, mM)	1	2	3

Table S2. Design of experiments using the CCD method to optimize niosomal formulations containing 5-FU.

Run	Independent variables		Dependent variables			
	Span® 60 (mM)	Cholesterol (mM)	Average size (nm)	PDI	Entrapment efficacy (%)	Release (%, 24 h)
1	6	3	324.7	0.285	67.39	52.81
2	4	3	267.5	0.257	62.21	47.07
3	6	1	251.3	0.335	73.25	39.92
4	2	2	197.5	0.214	79.55	41.39
5	6	2	235.9	0.274	85.58	65.37
6	4	2	225.3	0.254	83.21	59.21

7	2	3	240.8	0.227	56.75	51.52
8	2	1	174.2	0.305	62.43	28.27
9	4	2	214.8	0.237	80.90	62.44
10	4	1	185.3	0.270	75.22	34.38
11	4	2	211.7	0.238	78.39	63.72

Table S3. ANOVA statistical analysis for the quadratic polynomial model for particle size, PDI, entrapment efficacy and release.

**Source**	**F-Value**	**p-value**	
	**Particle size (nm)**		
Model	15.97	0.0043	
A	31.69	0.0025	Significant
B	39.36	0.0015	
	**PDI**		
Model	7.57	0.0222	
A	12.31	0.0171	Significant
B	11.17	0.0205	
B^2	9.23	0.0288	
	**Entrapment efficacy (%)**		
Model	15.67	0.0045	

A	11.35	0.0199	Significant
B	9.05	0.0298	
B^2	51.41	0.0008	
	**Release (%)**		
Model	7.01	0.0260	
B	10.11	0.0245	Significant
B^2	15.23	0.0114	

Table S4. Predicted models of 5-FU loaded niosomes.

**Models**
**Particle Size (nm) =** +212.65+33.23 * A+ 37.03* B +1.70* A * B + 10.97 * A2+ 20.67 * B2
**PDI =** +0.240 + 0.025 * A- 0.024* B + 7.000E-003* A * B +0.013 * A2+ 0.033 * B2
**Entrapment efficacy (%) =** + 81.99 + 4.58* A – 4.09* B – 0.045* A * B -1.16 * A2 - 15.01 * B2
**Release (%, 24h) =** +59.51+ 6.15 * A + 8.14 * B -2.59* A * B -2.72 * A2- 15.37 * B2

Table S5. Summary of the results of regression analysis for the various parameters (size, PDI, Entrapment efficacy and release) employed for fitting to the quadratic model.

Response	R-square	Adjusted R- square	Adequate precision	Lack of fit
Particle size	0.9411	0.8821	13.1600	0.1422
PDI	0.8834	0.7667	7.7600	0.1783
Entrapment efficacy (%)	0.9400	0.8800	11.4680	0.2970
Release (%)	0.8130	0.7502	8.295	0.0314

Table S6. Kinetic release models and parameters obtained for optimum niosomal formulation.

Kinetic Model		Zero-Order	First-Order	Higuchi	Korsmeyer-Peppas	
		$C_t=C_0+K_0t$	$LogC=LogC0+K_t/2.303$	$Q=K_H\sqrt{t}$	$M_t/M_\infty=Kt.t^n$	
		r^2	r^2	r^2	r^2	n*
5-FU (aq)	pH 7.4	0.7161	0.9695	0.9209	0.8856	0.8102
Nio/5-FU	pH 7.4	0.7304	0.8244	0.9649	0.9047	0.4352
	pH 5.4	0.7240	0.8611	0.9754	0.9116	0.3868
Nio/5-FU/PEG	pH 7.4	0.7284	0.7763	0.9340	0.9090	0.5171
	pH 5.4	0.7395	0.8156	0.9408	0.9329	0.4685
Nio/5-FU/FA	pH 7.4	0.8468	0.9215	0.9452	0.9374	0.4750
	pH 5.4	0.7291	0.8290	0.9571	0.9166	0.4210
Nio/5-FU/HA	pH 7.4	0.7694	0.8023	0.8880	0.9470	0.6439
	pH 5.4	0.7781	0.8393	0.9157	0.9529	0.5490

Table S7. Physical stability of Nio/5FU, Nio/5-FU/PEG, Nio/5-FU/HA and Nio/5-FU/FA (Mean ±SD, n = 3).

Sample	Storage temperature (^oC)	Storage time (day)	Size (nm)	PDI	Entrapment efficacy (%)
Nio/5-FU	4 ^oC	0	171.9 ±6.332	0.217±0.015	78.24±0.69
		30	242.75±8.95	0.284±0.013	74.91±1.38
	25 ^oC	0	171.9 ±6.332	0.217±0.015	78.24±0.69

		30	277.91±7.55	0.335±0.022	69.21±1.67
Nio/5-FU/PEG	4 ºC	0	105.4±5.36	0.184±0.007	86.91±1.63
		30	182.29±6.24	0.211±0.005	83.27±1.19
	25 ºC	0	105.4±5.36	0.184±0.007	86.91±163
		30	197.75±7.39	0.251±0.012	79.85±1.34
Nio/5-FU/FA	4 ºC	0	196.3±8.5	0.197±0.009	82.35±1.22
		30	241.92±77.62	0.242±0.013	78.63±1.75
	25 ºC	0	196.3±8.5	0.197±0.009	82.35±1.22
		30	265.84±9.5	0.295±0.014	72.21±1.38
Nio/5-FU/HA	4 ºC	0	223±10.54	0.219±0.01	78.24±0.69
		30	281.3±8.92	0.266±0.017	76.29±1.37
	25 ºC	0	223±10.54	0.219±0.01	78.24±0.69
		30	310.48±11.22	0.313±0.015	72.15±2.21
